# Nonlinearity Analysis and Parameters Optimization for an Inductive Angle Sensor

**DOI:** 10.3390/s140304111

**Published:** 2014-02-28

**Authors:** Lin Ye, Ming Yang, Liang Xu, Xiaoqi Zhuang, Zhaopeng Dong, Shiyang Li

**Affiliations:** Department of Instrument Science and Engineering, Shanghai Jiaotong University, Shanghai 200240, China; E-Mails: linye@sjtu.edu.cn (L.Y.); seablueboy@163.com (L.X.); qiqi_zxq@163.com (X.Z.); dzp0826@163.com (Z.D.); shiyangli@sjtu.edu.cn (S.L.)

**Keywords:** finite element method (FEM), particle swarm optimization (PSO), inductive angle sensor, nonlinearity error

## Abstract

Using the finite element method (FEM) and particle swarm optimization (PSO), a nonlinearity analysis based on parameter optimization is proposed to design an inductive angle sensor. Due to the structure complexity of the sensor, understanding the influences of structure parameters on the nonlinearity errors is a critical step in designing an effective sensor. Key parameters are selected for the design based on the parameters' effects on the nonlinearity errors. The finite element method and particle swarm optimization are combined for the sensor design to get the minimal nonlinearity error. In the simulation, the nonlinearity error of the optimized sensor is 0.053% in the angle range from −60° to 60°. A prototype sensor is manufactured and measured experimentally, and the experimental nonlinearity error is 0.081% in the angle range from −60° to 60°.

## 1. Introduction

Inductive position sensors are widely used in modern automotive and industrial applications [1–3]. They have various benefits such as low cost, good insensitiveness against temperature, and no wear-out [4–6]. Several types of position sensors based on the inductive principle differ in their nonlinearity errors [7]. The grating eddy current position sensor not only has the function of resisting liquids, but it also prevents ferromagnetic particles from affecting measurement results. However, measurement blind areas are not completely eliminated, so the linearity of the sensor is not satisfactory [8,9]. Inductive angle sensors are not susceptible to background electromagnetic interference, and they produce much greater output signal levels compared to other choices. However, there are usually higher order harmonic signals which lead to a considerable amount of nonlinearity errors [10]. Inductive angle sensors provide a compact structure and a high degree of insensitivity to production and installation tolerances, but the weak linear relationships between position and output signal (near the zero crossings) often lead to significant nonlinearity errors for calculating the angular displacement [11,12]. In the inductive position sensor field, the nonlinearity error is around one percent [7,13,14]. To reduce the nonlinearity error, the sensor structure needs to be optimized.

We previously presented an inductive angle sensor optimized using response surface methodology [15]. For simplicity the original paper did not discuss the influence of the sensor stator on the nonlinearity errors. However, it is found that the stator affects the behavior of electromagnetic fields within its rotor, which plays a key role in the linearity of the inductive angle sensor. Moreover, response surface methodology (RSM) has the following drawbacks: (i) RSM is strict in setting the initial search domain of the parameters. In a case where it is unsuitable, all experiments will have to be repeated; (ii) RSM is supposed to be a continuous optimization method, since RSM is similar to gradient-based approaches. Hence, unlike other optimizations, RSM is not suitable for discrete optimization; (iii) RSM may find a local optimum, as opposed to other optimizations that search for a global one [16]. On the other hand particle swarm optimization (PSO) doesn't readjust the initial search domain of the parameters [17]. PSO approaches are proposed for continuous and discrete optimization problems [18]. PSO is a member of the wide category of swarm intelligence methods for solving global optimization problems [19]. Compared with the design optimization of inductive sensor using genetic algorithms [20], PSO has no overlapping and mutation calculations with much simpler implementation.

In this paper, most parameters of the sensor are discussed, but understanding the parameters' effect on the nonlinearity error is a critical step in designing an effective sensor. Key parameters are chosen on the basis of their influence on the nonlinearity error. The finite element method and particle swarm optimization (PSO) are combined to design the sensor to achieve the minimum nonlinearity error.

This paper is organized as follows: in Section 2, the principle of the inductive angle sensor is described. In Section 3, key parameters for the design are selected and the sensor is optimized using PSO-FEM. The results are measured and discussed in Section 4. Finally, our conclusions about the sensor design is drawn in Section 5.

## 2. Principle of the Inductive Angle Sensor

**Figure 1.**
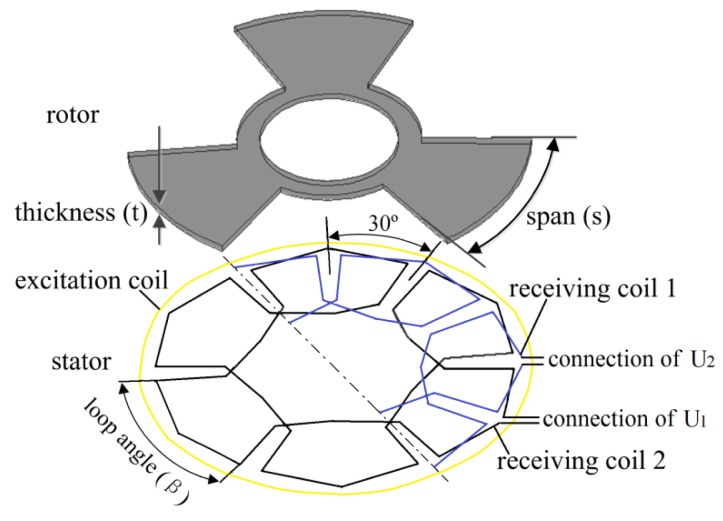
View of inductive angle sensor.

The proposed inductive angle sensor consists of a stator and a rotor, as illustrated in Figure 1. The stator has two receiving coils and one excitation coil, and the separation angle between the receiving coil 1 and 2 is 30°. The receiving coil comprises six loops with the same geometric shape. Adjacent loops are wound in the opposition direction. The stator layout has two advantages. The induced voltages in two receiving coils will be periodic when the rotor rotates. The induced voltages in two receiving coils are zero from the excitation coil because adjacent loops are symmetrical and wound in the opposition direction. However, the number of turns in two receiving coils is limited by the number of printed circuit board (PCB) layers. The multi-layer PCB layout will increase the cost burden. The number of turns in two receiving coils is a compromise between the performance and cost of the sensor.

A sine-wave voltage is applied to the excitation coil which generates an alternating magnetic field B_E_. The alternating magnetic field B_E_ induces an eddy current in the rotor, and the current creates an alternating magnetic field B_R_ that opposes the alternating magnetic field B_E_. The induced voltage in the receiving coil is shown in Equation (1) from the overlapping alternating magnetic fields B_E_ + B_R_. As is the symmetrical geometry of the receiving coil shown here, the portion of the induced voltage caused by the excitation coil (B_E_) is zero. Thus, only a voltage induced by the rotor (B_R_) remains in Equation (2):

(1) $$U_{O}=\frac{d\Phi }{dt}=\frac{d\int \left({\text{B}}_{E}\left(\text{t},\text{x},\text{y},\text{z}\right)+{\text{B}}_{R}\left(\text{t},\text{x},\text{y},\text{z}\right)\right)\text{dA}}{dt}$$

(2) $$U_{O}=\frac{d\int B_{R}\left(t,x,y,z\right)dA}{dt}$$

where *A* represents a surface area of the receiving coil.

**Figure 2.**
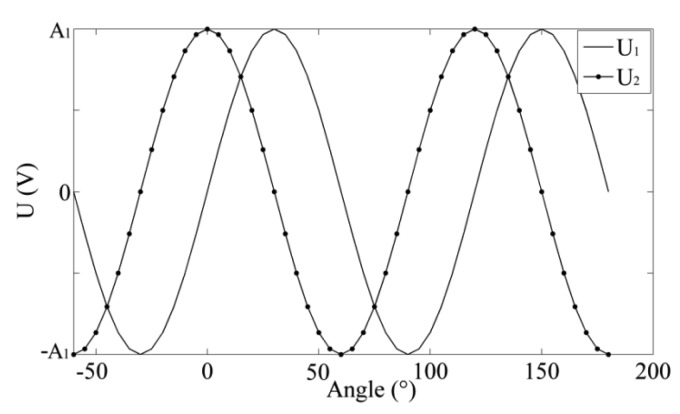
Induced voltages in two receiving coils.

When the rotor rotates, the change in coupling area between the rotor and the receiving coil will result in the variation of the induced voltage in the receiving coil. In a 120° cycle, the induced voltage in the receiving coil varies from zero to the maximum value in the negative direction, to zero, to the maximum value, and then to zero again. The induced voltage curve U_1_ in the receiving coil 1 approximately approaches the sinusoidal curve [21–23] in Figure 2. Due to a separation angle of 30° between the receiving coil 1 and 2, phase difference between induced voltages in two receiving coils is 90°. The induced voltage curve U_2_ in the receiving coil 2 draws close to the cosine curve. The curves U_1_ and U_2_ can be roughly expressed as:

(3) $$U_{1}=A_{1}\sin\frac{2\pi }{\lambda }\theta$$

(4) $$U_{2}=A_{1}\cos\frac{2\pi }{\lambda }\theta$$

Each cycle λ extends circumferentially over an angle of approximately 120°. When the rotor rotates the angle displacement Ψ, the induced voltage in the receiving coil will vary repetitively. Then the angle displacement Ψ can be written as:

(5) Ψ=mλ+θ

where Ψ denotes the angle displacement of the rotor, m represents the number of the complete cycles, and θ is the small angle displacement in one cycle λ.

Let the phase angle be:

(6) φ=2πθ/λ

Then:

(7) $$\varphi =\text{ATAN}2\left(U_{2},U_{1}\right)$$

**Figure 3.**
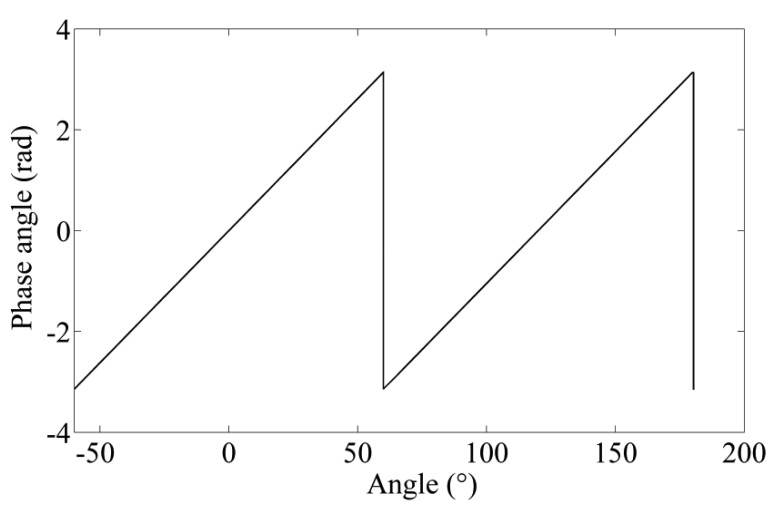
Linear phase angle changes *vs.* angle displacement.

It can be seen that the phase angle *φ* is proportional to the angle displacement in one cycle in Figure 3.

Thus the small angle *θ* mentioned can be obtained through the phase angle *φ*:

(8) θ=λφ/(2π)

Assuming *m* (the number of the cycles) is known, angle displacement can be calculated by Equation (5).

The linear relationship between the phase angle and angle displacement in one cycle is obtained on the basis of the assumption that induced voltage curves are ideal sinusoidal and cosine curves. However, this relationship is nonlinear due to the nonlinearity of the eddy current effect and systematic errors in the manufacturing and assembly processes.

The nonlinearity error [8,12] of the inductive angle sensor can be expressed in a measurement cycle:

(9) $$L=\frac{{\left|\varphi_{m}-\varphi_{i}\right|}_{\max}}{2\pi }\times 100\%$$

where *L* is the nonlinearity error of the inductive angle sensor, *φ_m_* is simulation phase angle or measured phase angle, and *φ_i_* is the idealized phase angle.

From the above analysis, the nonlinearity error of the sensor is affected by the stator and the rotor, which include the coil turn number, width of the coil in the stator, the loop angle, the rotor thickness, and the rotor blade span in the sensor. For the sake of simplicity, key variables are selected for the design of the sensor.

## 3. Optimization of Sensor Design

### 3.1. Analysis Setup of the Optimization

In order to model inductive angle sensor, the rotor and the stator are simulated in 3D. The parameters of the stator, the variables of the rotor, and even the material used in each component should be modeled exactly.

Besides geometrical modeling of sensor components, the excitation signal is set as 10×sin(2×*pi*×1,000,000×*time*). In general, the parts of the model are required to set boundary conditions according to their electromagnetic properties and structures. Initially, object interfaces are natural boundaries; outer boundaries and excluded objects are Neumann boundaries. Transient solver has been considered for this simulation. In mesh operations, there are meshing on object faces and meshing inside objects. Object faces have a maximum length of elements that is 1 mm. Inside objects have a maximum length of elements that is 2 mm.

In order to determine induced voltage in the receiving coil under the rotor rotation, 3D simulation has been performed in Ansoft-MAXWELL v.14. Solution setup should be determined and the problem should be solved. Therefore, the first step is to assign the kind of simulation solver for which Transient solver has been considered. Moreover, inductive angle sensor components should be meshed before solving the problem. Mesh operation has been done in the rotor, two receiving coils and the excitation coil. Also, this step has been done in 10 passes, which are initialized with 1% error. In addition, the increasing grate of meshing has been considered as 5% per pass. Based on this procedure, the total number of meshed elements in the sensor is about 993,334. The number of meshed elements in each component has been listed in Table 1.

**Figure 4.**
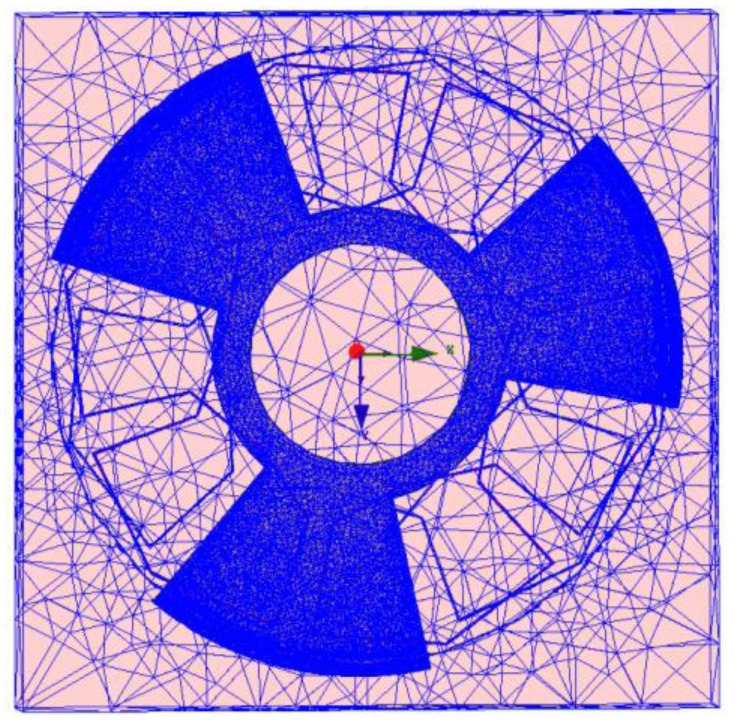
3D modeling of studied sensor under mesh operation.

Also, a 3D meshed model of studied sensor has been shown in Figure 4. It should be noted that in 3D simulations, all meshes have tetrahedral shapes. It should also be mentioned that for all simulations of this paper, time step has been chosen about 1e–008 s.

### 3.2. Sensor Parameter Selection

Several variables in the sensor are listed in. The sensor parameters include coil turn number, width of the coil in the stator, the loop angle, the rotor thickness, and the rotor blade span. According to technological requirements and theoretical deduction, the initial values and setting range of parameters are set as shown in Table 2. In this design, the gap between the stator and the rotor is 0.2 mm in consideration of electromagnetic intensity and process limits.

#### 3.2.1. Coil Turn Number

The excitation coils with various numbers of turns are designed and simulated, and the induced voltages in the sensor are generated. Values in Table 3 show the maximum error between simulation values and theoretical values in the −50° and 20° range is ±0.037, which suggests that the nonlinearity error of the sensor with a three turn excitation coil is 0.589% in the range of ±60° according to Equation (9). The number of turns in the excitation coil is changed from 3 to 11, and the nonlinearity error has a variation of almost 0.021% according to Table 4.

#### 3.2.2. Coil Width

Based on the Inductance Calculations Manual [24], the coil width has little effect on coil inductance and impedance. The coil width is changed from 0.1 mm to 0.8 mm, and the nonlinearity error has a variation of 0.044% as seen in Table 5.

#### 3.2.3. Loop Angle

There are six loops in the receiving coil and three rotor blades in the rotor in Figure 1. The coupling area between the rotor and the receiving coil is changed to produce the induced voltage in the receiving coil. Loop angle in the receiving coil has influence on the induced voltage when the rotor rotates. In this design, loop angle range is designed from 45° to 57.5°. After the simulations are executed, the induced voltages in the sensor are generated. Nonlinearity errors of the sensors with different loop angles are calculated in Equation (9). The nonlinearity error has a variation of 0.103% as shown in Table 6.

#### 3.2.4. Rotor Thickness

The rotor thickness affects the intensity of the current in the rotor, which influences the induced voltage in the receiving coil. In Table 7, the rotor thickness changes from 0.5 mm to 1.75 mm, and the nonlinearity error has a variation of almost 1%.

#### 3.2.5. Spans of the Rotors Blades

The coupling area between the rotor and the receiving coil is adjusted to produce the induced voltage in the receiving coil when the rotor rotates. In Table 8, the span of the rotor blade is designed from 45° to 57.5°, and the nonlinearity error has a variation of 0.42%.

To sum up, the nonlinearity error has a variation of almost 1% when the rotor thickness changes from 0.5 mm to 1.75 mm; a variation of 0.42% in the nonlinearity error is achieved as the span of the rotor blade varies from 45° to 57.5°. Nonlinearity error has a variation of less than 0.12% in the change of other parameters. The rotor parameters are key factors for the sensor design.

### 3.3. Optimization of the Sensor

In order to optimize the sensor design and reduce the nonlinearity error, an optimization design method using PSO and FEM is proposed to determine the optimal dimensions of the sensor. Particle swarm optimization consists of a swarm of particles. Each particle has a position that represents a possible solution within the search space, and a velocity. PSO is used to search for the optimal design variables by minimizing the fitness function among the feasible region. Inductive angle sensor is designed based on design variables and simulated by finite element method, which can calculate the nonlinearity error. In PSO–FEM design method, the nonlinearity error is formulated as a fitness function; Finite element method, in which the rotor parameters are adopted as the design variables, is used as a solver of the fitness function. Then, the search for the optimal dimensions of the rotor is converted to the search for the optimal particle.

In PSO–FEM, each particle has a position $x_{i}=(x_{i}^{1},x_{i}^{2},\ldots ,x_{i}^{n})$ and a velocity $v_{i}=(v_{i}^{1},v_{i}^{2},\ldots ,v_{i}^{n})$ in the n-dimensional problem space [25]. Where *i* denotes the *ith* particle and *n* denotes the number of optimized parameters. When the PSO optimizer is started, a swarm of particles are initially placed at random positions in the search-space and moving in randomly defined directions. In each step of updating iteration, PSO sends each particle, which represents a particular structure dimension of the rotor to the FEM simulation and calculates the nonlinearity error using the simulation results. The personal best position (denoted by *p_i_*) and the global best position (denoted by *p_g_*) are updated on the basis of the fitness values. Then, new velocity and new position of each particle are updated according to the following Equations (10) and (11):

(10) $$v_{i}\;(t+1)=\omega \;v_{i}\;(t)+c_{1}r_{1}\;\left(p_{i}(t)-x_{i}\;(t)\right)+c_{2}r_{2}\;\left(p_{g}\;(t)-x_{i}\;(t)\right)$$

(11) $$x_{i}\;(t+1)=x_{i}\;(t)+v_{i}\;(t+1)$$

with inertia weight, *ω* acceleration coefficient *c*_1_and *c*_2_, and random vector *r*_1_, *r*_2_.

**Figure 5.**
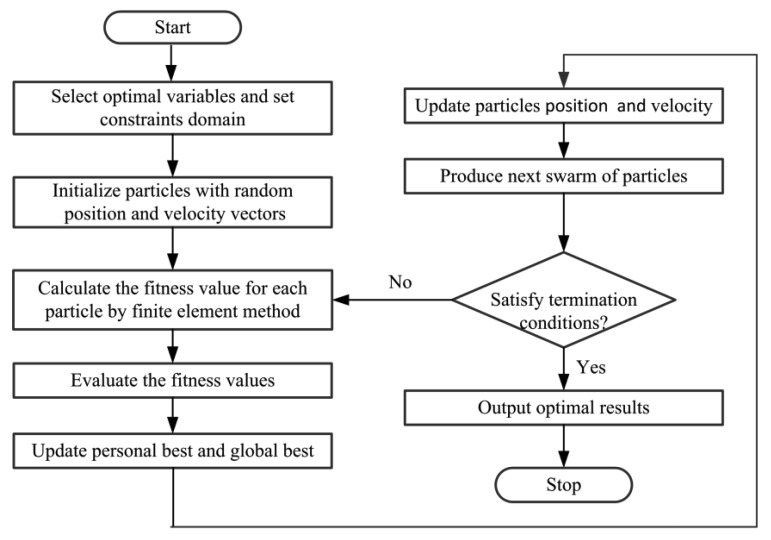
Flow chart of optimization design using PSO–FEM.

This iteration process continues until all particles convergence the same solution or total generation number is reached. The flow chart of optimization design is shown by PSO–FEM in Figure 5. In this way, an optimal design of the sensor can be achieved.

Since the structure of the rotor is simple, thickness of the rotor (t) and span of the rotor blade (s) are selected as optimal variables in Figure 1. The optimal design of the sensor can be transformed to the problem of determining the values of design variables by minimizing nonlinearity error. Then, the mathematical model for the optimal rotor design is minimizing the fitness function with the constrained condition and can be expressed as:

(12) $$\text{fitness}=L=\frac{{\left|\varphi_{m}-\varphi_{i}\right|}_{\max}}{2\pi }\times 100\%$$

Subject to: 0.5*mm*≤*t*≤2*mm*

45°≤s≤60°

where L is the nonlinearity error of the inductive angle sensor, *φ_m_* is simulation phase angle or measured phase angle, and *φ_i_* is the idealized phase angle.

**Figure 6.**
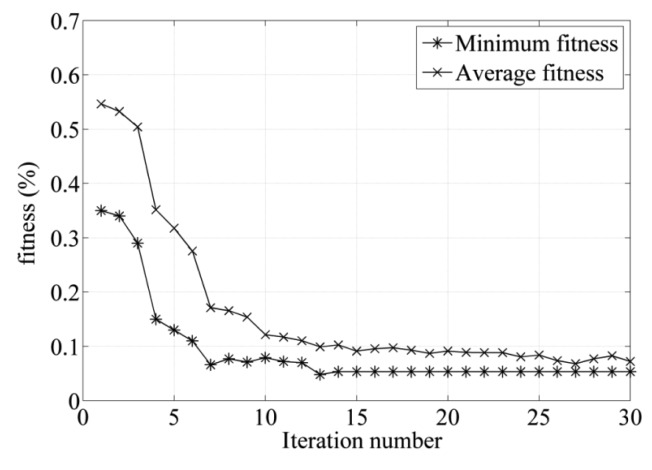
Variation of fitness value with iteration numbers.

In the optimization design for the sensor using PSO–FEM method, the number of the particles is set as 20. The number of optimized parameters is taken as 2. A constant value of the inertia weight *ω* = 0.7, and acceleration coefficients *c_1_* = *c_2_* = 0.65 are chosen. Using this PSO–FEM approach, the parameters of the rotor are optimized to get minimum fitness value and the optimal structure of the rotor can be found. The iteration process of the fitness function is shown in Figure 6, and when the iteration number exceeds 15, the minimum and average fitness values reach the stable values 0.06% and 0.1%, respectively.

**Figure 7.**
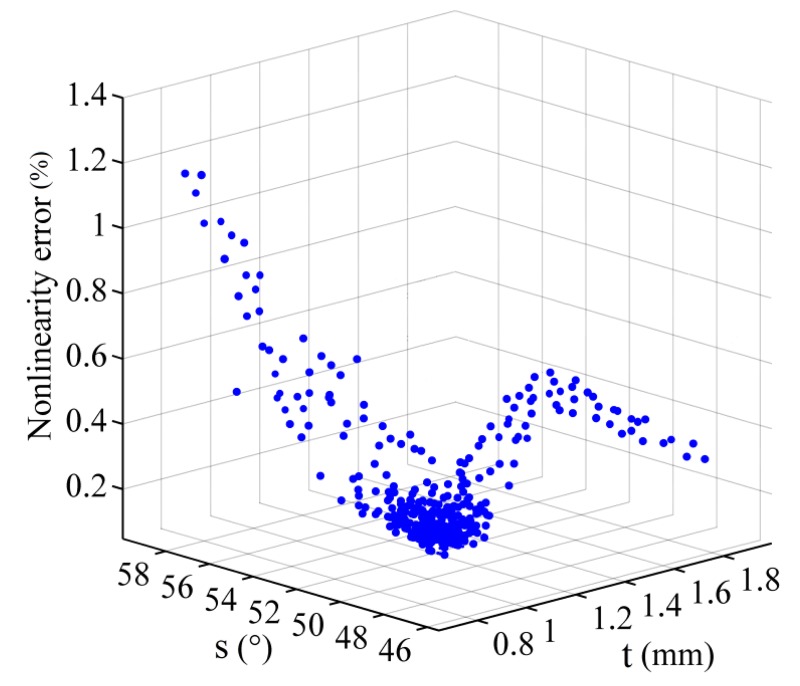
Nonlinearity error *vs.* the corresponding thickness and span obtained in the optimization design.

**Figure 8.**
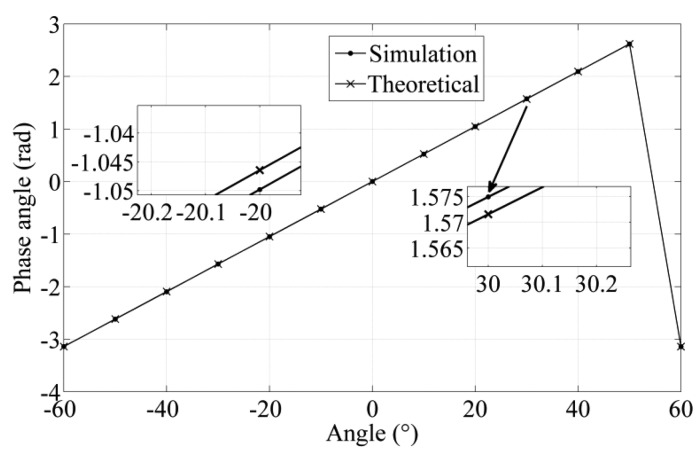
Error of the simulation model in phase angle.

After the iteration process is completed, the nonlinearity error, the corresponding thickness, and span of the rotor blade obtained in optimization process are plotted in Figure 7. The nonlinearity error is affected by the combination of thickness and the span of the rotor blade. When the thickness of the rotor approximates 0.5 mm, the nonlinearity error is about 1%. The optimization results of the sensor and corresponding parameters are shown in Table 9. The rotor model is designed based on a rotor thickness of 1.24 mm and a rotor blade span of 52.7°. The partial enlarged detail of phase angle shows maximum errors of ±0.0033 between simulation value and theoretical value in −20° and 30° in Figure 8, so the nonlinearity error is 0.053% on the basis of Equation (9).

## 4. Fabrication and Test of the Sensor

**Figure 9.**
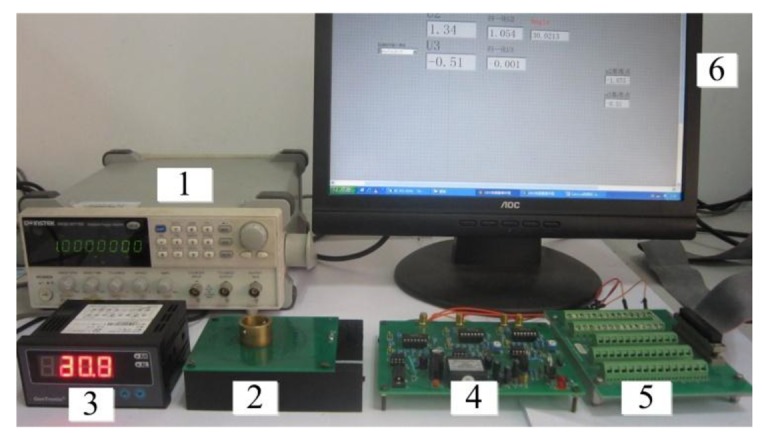
Configuration of the experimental setup.

**Figure 10.**
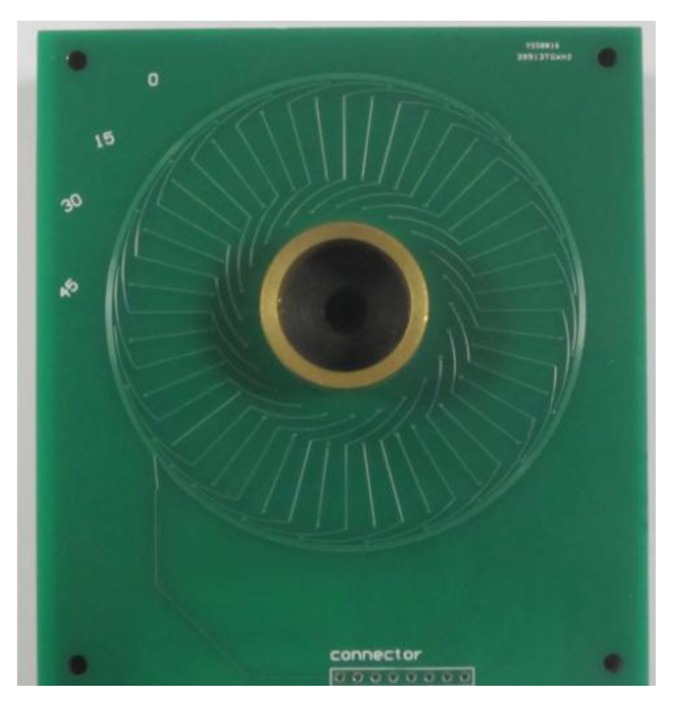
Enlarged view of the manufactured angle sensor.

To complete the research, the sensor is designed using optimal parameters. The experimental configuration is depicted in Figure 9, which illustrates signal generator, inductive angle sensor, signal process board, display device, Labview DAQ board and monitor. The rotor is fabricated with copper material and the coils of the stator are designed on the top and bottom layers of PCBs as shown in Figure 10. When the rotor rotates, its position signal modulates the excitation signal, which comes from signal generator. To get the position of the rotor, the signals in two receiving coils needs to be demodulated in signal process board. The demodulated signals are then sent to the Labview DAQ board.

**Figure 11.**
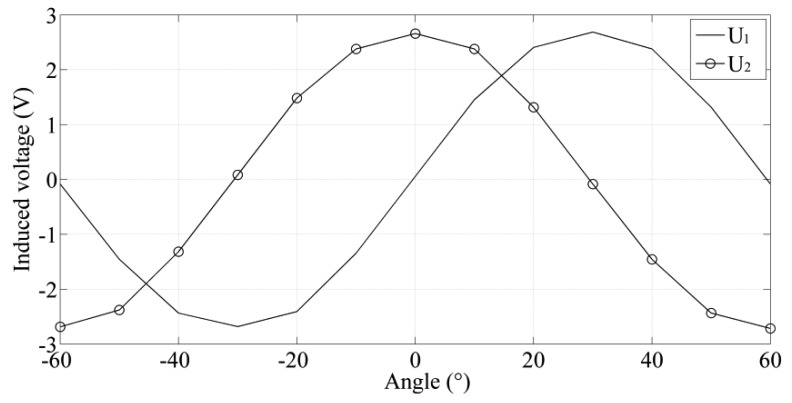
Demodulated signals in two receiving coils.

**Figure 12.**
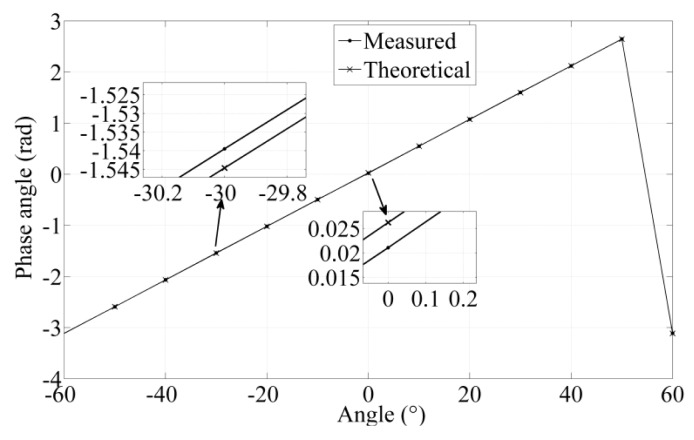
Error of the sensor prototype in phase angle.

When the rotor rotates, a set of induced voltage values in two receiving coils are recorded in Figure 11. Based on these induced voltage values, the position of the rotor represented with the angle is calculated according to Equation (7). In Figure 12, the experimental phase angle is measured by the designed sensor and the theoretical phase angle is measured by the contact angle instrument. The partial enlarged detail of phase angle shows maximum errors of ±0.0051 between measured value and theoretical value in −30° and 0° respectively, which suggests that the nonlinearity error is 0.081% on the basis of Equation (9).

While the rotor is rotating, the induced voltage in the receiving coil will vary repetitively. The sensor has an infinite measurement range. Many experiments with a prototype sensor are executed to repeat the measurement of the nonlinearity error, 0.081%. The experiments demonstrate the accuracy of ±0.15° and a sensitivity of 0.08° in full scale.

## 5. Conclusions

A contactless inductive angular-position sensor has been designed, built, and tested. The sensor is made up of an excitation coil, two receiving coils and a rotor. The induced voltages in the receiving coils vary in a sinusoidal way with the change of rotor angular position related to the stator. The sensor is simulated using Ansoft's Maxwell software. For the sake of simplicity, key parameters are selected for the design based on the parameters' effect on the nonlinearity errors. The finite element method and particle swarm optimization are combined for the sensor design minimize the nonlinearity error. The simulation results indicate that the optimized sensor has a nonlinearity error of 0.053%. To verify the validity of the design, a prototype sensor has been fabricated, and the experimental nonlinearity error is 0.081%.

## Figures and Tables

**Table 1.** Mesh operation information of studied sensor in different components.

**Parts**	**Number of Mesh Components**
Excitation coil	22,395
Receiving coil 1	5,919
Receiving coil 2	5,814
Region	517,465
Rotor	441,741
Total	993,334

**Table 2.** Input parameters of sensor model.

**Abbreviation**	**Meaning**	**Initial Value**	**Setting Range**
*N_1_*	Excitation coil turn number	3	3–11
*W*	Width of the coil	0.1 mm	0.1 mm–0.8 mm
*β*	Loop angle	52.5°	45°–57.5°
*t*	Thickness of the rotor	0.75 mm	0.5 mm–2 mm
*s*	Span of the rotor blade	57.5°	45°–57.5°

**Table 3.** Nonlinearity error of the sensor with three turns of the excitation coil.

***R_P_ (°)***	***U_1_ (mv)***	***U_2_ (mv)***	***φ_m_***	***φ_i_***	***φ_m_*−*φ_i_***	***L***
−60	3.6097	−662.258	3.1361	3.1325	0.0036	0.057%
−50	−322.699	−524.653	−2.5902	−2.6272	0.0370	**0.589%**
−40	−518.917	−329.784	−2.1369	−2.1035	−0.0334	−0.532%
−30	−647.948	−1.8668	−1.5737	−1.5799	0.0062	0.099%
−20	−523.683	318.128	−1.0249	−1.0563	0.0314	0.5%
−10	−326.258	510.554	−0.5686	−0.5327	−0.0359	−0.572%
0	−2.4504	649.71	−0.0038	−0.0091	0.0053	0.084%
10	324.429	531.821	0.5478	0.5145	0.0333	0.53%
20	515.065	329.886	1.0011	1.0381	−0.037	**−0.589%**
30	656.651	−0.8351	1.5721	1.5617	0.0104	0.166%
40	525.549	−321.415	2.1197	2.0853	0.0344	0.548%
50	329.475	−561.811	2.6112	2.6089	0.0023	0.037%
60	1.7631	−657.818	3.1389	3.1325	0.0064	0.102%

R_P_—Rotor position.

**Table 4.** Nonlinearity errors of the sensors with different coil turn numbers.

***N_1_***	***W***	***β***	***t***	***s***	***L***	***E_V_***
3	0.1 mm	52.5°	0.75 mm	57.5°	**0.589%**	0.021%
5	0.1 mm	52.5°	0.75 mm	57.5°	0.594%	
7	0.1 mm	52.5°	0.75 mm	57.5°	0.605%	
9	0.1 mm	52.5°	0.75 mm	57.5°	**0.610%**	
11	0.1 mm	52.5°	0.75 mm	57.5°	0.607%	

E_V_—error variation.

**Table 5.** Nonlinearity errors of the sensor with different coil widths.

***N_1_***	***W***	***β***	***t***	***s***	***L***	***E_V_***
3	0.1 mm	52.5°	0.75 mm	57.5°	**0.589%**	0.044%
3	0.2 mm	52.5°	0.75 mm	57.5°	0.604%	
3	0.5 mm	52.5°	0.75 mm	57.5°	**0.633%**	
3	0.8 mm	52.5°	0.75 mm	57.5°	0.615%	

E_V_—error variation.

**Table 6.** Nonlinearity errors of the sensors with different loop angles.

***N_1_***	***W***	***β***	***t***	***s***	***L***	***E_V_***
3	0.1 mm	45°	0.75 mm	57.5°	**0.549%**	0.103%
3	0.1 mm	47.5°	0.75 mm	57.5°	0.561%	
3	0.1 mm	50°	0.75 mm	57.5°	0.573%	
3	0.1 mm	52.5°	0.75 mm	57.5°	0.589%	
3	0.1 mm	55°	0.75 mm	57.5°	0.635%	
3	0.1 mm	57.5°	0.75 mm	57.5°	**0.652%**	

E_V_—error variation.

**Table 7.** Nonlinearity errors of the sensors with different rotor thickness.

***N_1_***	***W***	***β***	***t***	***r***	***L***	***E_V_***
3	0.1 mm	52.5°	0.5 mm	57.5°	**1.128%**	0.935%
3	0.1 mm	52.5°	0.75 mm	57.5°	0.589%	
3	0.1 mm	52.5°	1 mm	57.5°	0.339%	
3	0.1 mm	52.5°	1.25 mm	57.5°	**0.193%**	
3	0.1 mm	52.5°	1.5 mm	57.5°	0.377%	
3	0.1 mm	52.5°	1.75 mm	57.5°	0.325%	

E_V_—error variation.

**Table 8.** Nonlinearity errors of the sensors with different spans of the rotors blades.

***N_1_***	***W***	***β***	***t***	***s***	***L***	***E_V_***
3	0.1 mm	52.5°	0.75 mm	45°	**0.692%**	0.42%
3	0.1 mm	52.5°	0.75 mm	47.5°	0.372%	
3	0.1 mm	52.5°	0.75 mm	50°	**0.272%**	
3	0.1 mm	52.5°	0.75 mm	52.5°	0.408%	
3	0.1 mm	52.5°	0.75 mm	55°	0.593%	
3	0.1 mm	52.5°	0.75 mm	57.5°	0.589%	

E_V_—error variation.

**Table 9.** Optimal variables for nonlinearity error.

**Optimal Variables**		**Nonlinearity Error (%)**	
t	s	L_1_	L_2_
1.24 mm	52.7°	0.053	0.081

L_1_—onlinearity error of simulation model, L_2_—onlinearity error of prototype sensor.
